# Improving Diabetes Equity and Advancing Care (IDEA) to optimize team-based care at a safety-net health system for Black and Latine patients living with diabetes: study protocol for a sequential, multiple assignment, randomized trial

**DOI:** 10.1186/s13063-024-08346-9

**Published:** 2024-07-24

**Authors:** Jacquelyn Jacobs, Patricia Labellarte, Helen Margellos-Anast, Lizbeth Garcia, Fares Qeadan, Benjamin Tingey, Kelsey Barnick, Alyn Dougherty, Christina Wagener

**Affiliations:** 1 Sinai Urban Health Institute, Sinai Health System, 1500 South Fairfield Avenue, Chicago, IL 60608 USA; 2 Parkinson School of Health Sciences and Public Health, Loyola University Chicago, 2160 S 1St Ave, Maywood, IL USA; 3 Center for Diabetes and Endocrinology, Sinai Health System, 1500 South Fairfield Avenue, Chicago, IL USA

**Keywords:** Diabetes, Diabetes self-management training, Community health worker, Remote glucose monitoring, Chronic disease, Sequential multiple assignment randomized trials (SMART), Health equity

## Abstract

**Background:**

Diabetes is the eighth leading cause of death in the USA. Inequities driven by structural racism and systemic oppression have led to racial/ethnic disparities in diabetes prevalence, diagnosis, and treatment. Diabetes-self management training (DSMT), remote glucose monitoring (RGM), and tailored support from a community health worker (CHW) have the potential to improve outcomes. This study will examine the implementation of these interventions in a safety-net healthcare setting.

**Methods:**

Using implementation science and racial equity principles, this study aims to (1) evaluate the appropriateness; (2) measure fidelity; and (3) compare the effectiveness of varying the combination and sequence of three interventions. An exploratory aim will measure sustainability of intervention adherence and uptake. This mixed-methods trial employs a sequential, multiple assignment randomized trial (SMART) design, patient focus group discussions, and staff interviews. Eligible Black/Latine patients will be recruited using patient lists extracted from the electronic medical record system. After a detailed screening process, eligible patients will be invited to attend an in-person enrollment appointment. Informed consent will be obtained and patients will be randomized to either DSMT or RGM. At 6 months, patients will complete two assessments (diabetes empowerment and diabetes-related distress), and HbA1c values will be reviewed. “Responders” will be considered those who have an HbA1c that has improved by at least one percentage point. “Responders” remain in their first assigned study arm. “Nonresponders” will be randomized to either switch study arms or be paired with a CHW. At 6 months participants will complete two assessments again, and their HbA1c will be reviewed. Twelve patient focus groups, two for each intervention paths, will be conducted along with staff interviews.

**Discussion:**

This study is the first, to our knowledge, that seeks to fill critical gaps in our knowledge of optimal sequence and combinations of interventions to support diabetes management among Black and Latine patients receiving care at a safety-net hospital. By achieving the study aims, we will build the evidence for optimizing equitable diabetes management and ultimately reducing racial and ethnic healthcare disparities for patients living in disinvested urban settings.

**Trial registration:**

ClinicalTrials.gov: NCT06040463. Registered on September 7, 2023.

**Supplementary Information:**

The online version contains supplementary material available at 10.1186/s13063-024-08346-9.

## Introduction

### Background and rationale {6a}

Diabetes is the eighth leading cause of death in the USA and can lead to heart disease, vision loss, kidney disease, and ultimately death [1]. The American Diabetes Association estimates the cost of diabetes due to treatment and lost productivity to be $327 billion in 2017, a 26% increase since 2012 [2]. Racial and ethnic diabetes prevalence, diagnosis, and treatment disparities are widespread and well-documented [3]. Health and social inequities, driven by structural racism and systemic oppression, lead to a lack of safe and secure housing, nutritious and accessible food, reliable transportation, and healthcare access, which can predict poor disease outcomes. In Chicago, 11.3% of adults are living with diabetes, with Black and Hispanic Chicagoans disproportionately affected [4]. In 2022, Hispanic individuals were nearly three times more likely and Black individuals more than twice as likely to have diabetes compared to their White counterparts (14.7%, 12.5% and 5.5%, respectively) [4]. Substantial disparities also exist in diabetes-related hospitalization; Hispanic individuals had double the rate and Black individuals almost four times the rate of diabetes-related hospitalizations compared to White individuals (25.3 per 10,000, 40.3 per 10,000 and 12.5 per 10,000, respectively) [5].

There are several evidence-based diabetes management practices such as dietary changes, increased physical activity, and lifestyle education programs. Team-based care (TBC) is one systems-level care model that has been shown to be effective in managing hemoglobin A1c (HbA1c), blood pressure, and cholesterol among patients with diabetes [6–8]. The Institute of Medicine defines TBC as one in which a patient’s care team include the patient, the patient’s primary care team, and at least one other health care provider [9]. While randomized controlled trials and meta-analyses have demonstrated the effectiveness of TBC, less is known about the impact of particular TBC enhancements, the sequencing of those enhancements on outcomes, and their effectiveness among uninsured patients residing in historically disinvested communities [7]. Given the complex interplay of health and social determinants, there is no one-size-fits-all approach when it comes to addressing chronic diseases such as diabetes. Solutions must address all interrelated factors and consider the uniqueness of each patient.

Three interventions that support and/or teach diabetes management strategies and can ultimately reduce HbA1c are diabetes-self management training (DSMT), remote glucose monitoring (RGM), and tailored support from a community health worker (CHW). DSMT teaches individuals how to live with and manage a diabetes diagnosis and has been proven to increase participant lifestyle change, reduce blood glucose levels, HbA1c, and lipid profiles [10]. RGM is an automated process of transmitting blood glucose information directly to a health care provider using a mobile application. RGM relies on valid and reliable blood glucose measures which are measured by the patient outside of a healthcare setting. Measures come from a glucometer or insulin pump, which are regulated by the United States Food and Drug Administration (FDA). The FDA requires 95% of readings from all self-monitoring glucose devices to be accurate within 15–20% above or below the true reading [11, 12]. The literature on RGM is not extensive, but has shown promise for improving glycemic control [13–15]. Finally, CHWs are specially trained, trusted members of the community that have been integrated into healthcare settings to provide tailored support to patients related to social needs and reinforcing education and lifestyle changes [16].

#### Framework

This study is guided by implementation science and racial equity principles. Implementation science seeks to explore the systematic uptake or improvement of an evidence-based practice into real-world practice. Employing implementation science principles facilitates a deeper understanding of the challenges around successfully delivering innovations in real-world settings. In recent years, implementation science has become a frequently used way of thinking to improve policies and practices within health care settings. Similarly, the public health community, healthcare institutions, and policymakers have become more familiar with racial and health equity principles and their potential to reduce health and healthcare-related disparities among racial and ethnic minorities. Implementation science experts have called for health services research to prioritize health equity.

**Fig. 1:**
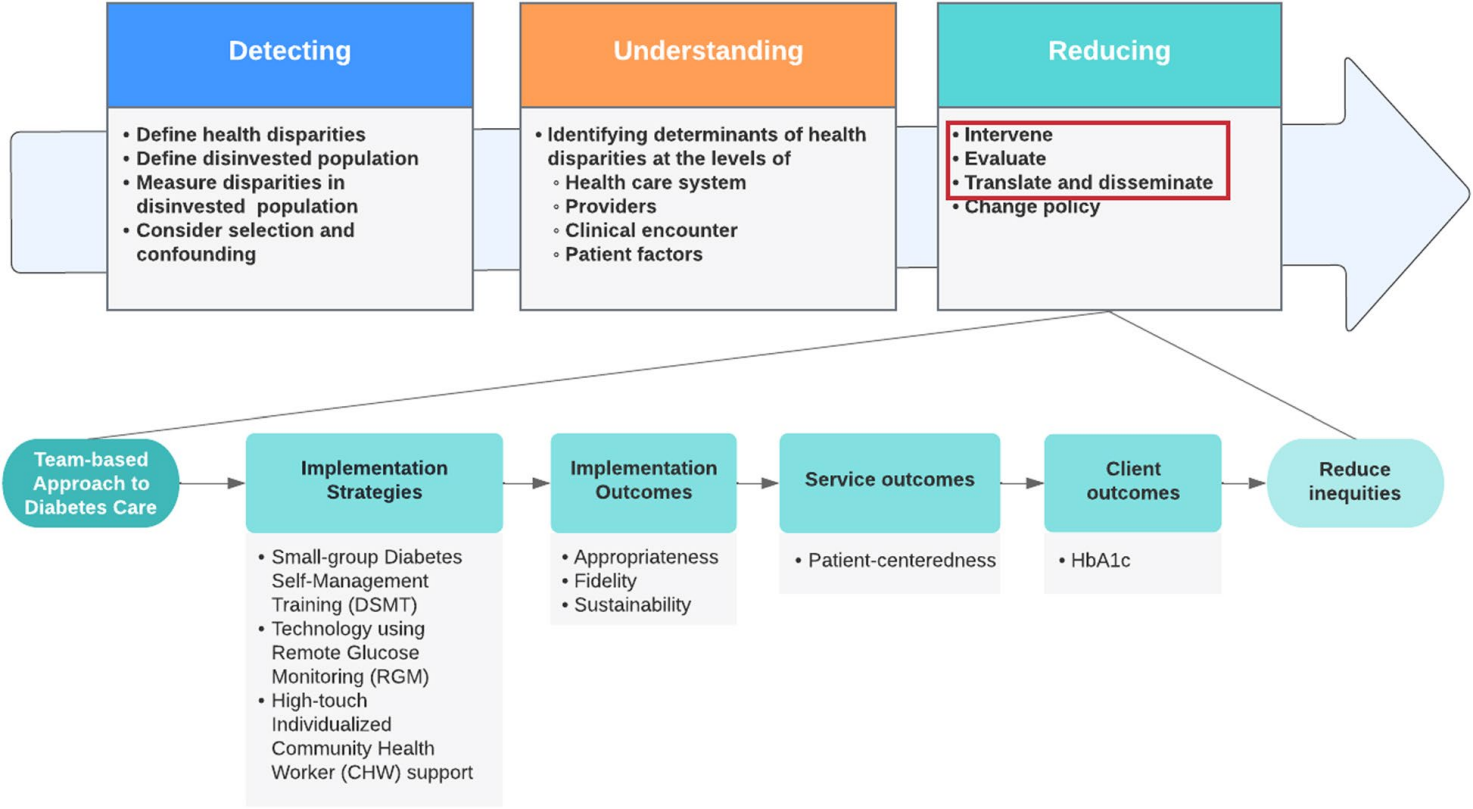
IDEA Study Conceptual Framework

This study integrates Proctor et al.’s Outcomes for Implementation Research framework and Kilbourne’s Advancing Health Disparities Research framework to present a conceptual model (Fig. 1. IDEA Study Conceptual Framework) for rigorous measurement of implementation, service, and patient outcomes with an equity lens [17, 18]. Kilbourne proposes three phases of advancing health disparities research: (1) *detecting* the health disparities and identifying the disinvested populations; (2) *understanding* the determinants of these health disparities at varying levels of our healthcare system; and (3) *reducing* disparities by intervening, evaluating, translating, and disseminating findings. The present study focuses on *reducing* disparities. The implementation outcomes of interest are *appropriateness* of each of the three interventions, *fidelity* to intervention adherence, and *sustainability* of disease management strategies. Appropriateness refers to the perceived fit or compatibility of these strategies for the patient population, their needs, and for the setting. Fidelity refers to the degree to which the strategy is implemented as intended. Sustainability refers to the extent to which the treatment is sustained or institutionalized.

### Objectives {7}

This study seeks to achieve the following aims:

- Aim 1: Evaluate the appropriateness of DSMT, RGM, and high-touch, individualized CHW support from the provider and patient perspectives.
- Aim 2: Measure fidelity of DSMT, RGM, and high-touch, individualized CHW support for Black/Latine patients with diabetes in a safety-net hospital setting.
- Aim 3: Compare the effectiveness of varying the sequence of three enhancements (DSMT, RGM, and high touch, individualized CHW support) to reduce HbA1c among Black/Latine patients with diabetes.
  - Exploratory Aim 3a: Measure the extent to which high-touch, individualized CHW support reinforces DSMT concepts and RGM uptake and adherence for Black/Latine patients with diabetes (sustainability).

### Trial design {8}

**Fig. 2:**
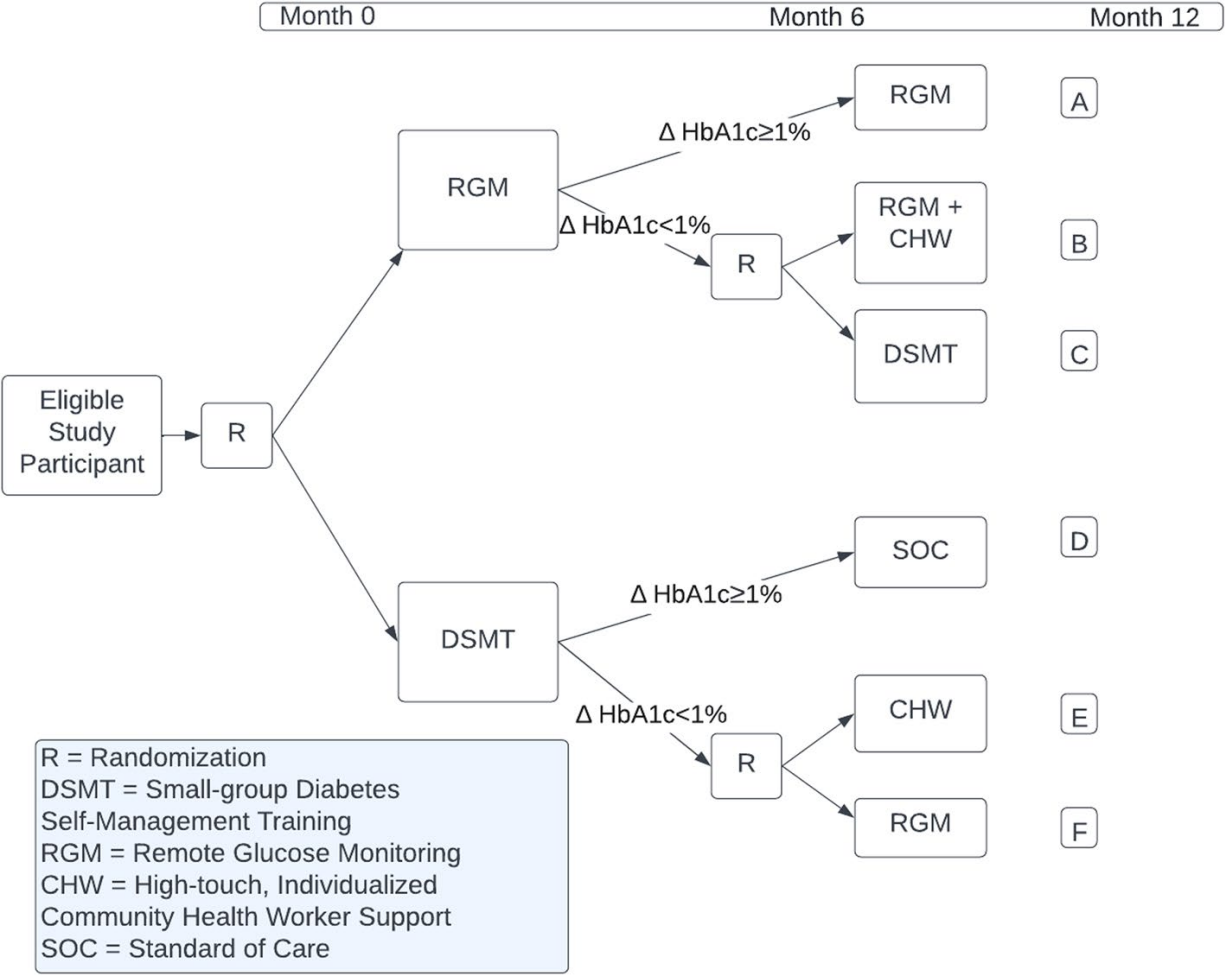
IDEA Study SMART Design

This study uses a sequential, multiple assignment, randomized trial (SMART) design with approximately 270 Black/Latine patients living with diabetes recruited from two locations of a diabetes and endocrinology clinic (“the Center”) in Chicago, IL. A SMART is a type of adaptive trial that uses at least two points of randomization to build the evidence for an optimal intervention or treatment. In the IDEA study SMART, we will test three components of a team-based care model that provides diabetes care and management for patients living with diabetes and other complex health and social needs. HbA1c will serve as our pre-planned tailoring variable which will determine participant rerandomization. At 6 months, participants with an HbA1c of at least one percentage point improvement from their baseline measurement will be considered “responders”; participants with an HbA1c that has not improved by at least one percentage point from their baseline measurement will be considered “non-responders”. The study will be conducted by Sinai Urban Health Institute (SUHI), the community-engaged research institute within Sinai Chicago, a safety-net health system in Chicago, IL. See Fig. 2. IDEA Study SMART Design, for the study design. We hypothesize that path E (DSMT to CHW) will have the largest reduction in A1c compared to all other paths. DSMT provides actionable education and guidance for improving diabetes management, whereas RGM focuses on information tracking for both patients and providers. The CHW will reinforce information taught during DSMT sessions.

## Methods: participants, interventions, and outcomes

### Study setting {9}

The study will take place at Sinai Chicago in Chicago, Illinois, USA. Sinai Chicago is the largest private safety-net health system in Illinois and includes three hospitals: Mount Sinai Hospital (MSH), Holy Cross Hospital (HCH), and Schwab Rehabilitation Hospital. More than 100,000 people with diabetes live in Sinai Chicago’s primary service area, and approximately 70% of all Sinai Chicago patients live with diabetes or prediabetes. Communities in Sinai Chicago’s primary service area have some of the most severe disparities related to diabetes incidence, control, and complications. In Mount Sinai Hospital’s 2022 Community Health Needs Assessment, diabetes was the fifth most important community health need according to more than 560 surveyed individuals in the service area [19]. Specifically, the study will take place at two Center sites, one at MSH and one at HCH. The Center was launched in September 2020 to treat patients with pre-diabetes, diabetes, and other endocrine disorders. The Center is a robust, patient-centered Diabetes Center of Excellence with coordinated care amongst several clinicians.

### Eligibility criteria {10}

Eligibility criteria for each study component (SMART, participant focus group discussions, and staff interviews) are described in Table 1.

**Table 1:** IDEA study eligibility criteria

Study component	Inclusion criteria	Exclusion criteria
SMART	• Diagnosed with type 1 or type 2 diabetes • 18 years of age or older • Able to provide informed consent • African American/Black or Latine • HbA1c level ≥ 7% measured within 365 days prior to study enrollment • Have a smartphone compatible with the Glooko RGM application [20] • Completed at least one appointment at the Center since the Center opened in 2020 • Fluent in English or Spanish	• Have an activated Power of Attorney • Diagnosed with Stage V renal disease or undergoing dialysis • Diagnosed with a severe form of cardiovascular disease • Diagnosed with gestational diabetes only (without type 1 or type 2) • Diagnosed with any severe form of mental disorder including schizophrenia, bipolar disorder, or severe depression • Pregnant • Diagnosed with Alzheimer’s disease or dementia • Already an active user of RGM • Actively working with a CHW for their diabetes • Planning to travel outside of the Chicagoland area for more than three months in the year following study enrollment • Use a continuous glucose monitor that is incompatible with the Glooko RGM application
Participant Focus Group Discussions	• Meet all eligibility for SMART component of study • Completed at least 3 months of their assigned SMART study condition	None
Center Staff Interviews	• Employed by the Center during the study period • 18 years of age or older • Able to provide informed consent • Fluent in English or Spanish	None

### Who will take informed consent? {26a}

Participants who are deemed eligible based on the above eligibility criteria, and agree to be randomized, will provide written informed consent before participating in the study. During the informed consent process, a member of the research team will explain the study in detail, including its objectives, the funding source, who is being invited to participate, what will take place in each study arm, the duration of the study, and how many people will be expected to participate. They will explain the process of randomization, the three interventions being tested (DSMT, RGM, and CHW support), potential risk and benefit, and how risk will be mitigated. The research specialist will answer all participant questions and obtain a signature if the participant is still interested in participating in the study. All participants who provide a signature on the consent form will receive a copy to take home. This will detail the study activities and contact information for the principal investigators (PIs) and who to contact at Sinai Chicago with any additional questions or concerns.

### Additional consent provisions for collection and use of participant data and biological specimens {26b}

Not applicable. All consent provisions have been described above.

## Interventions

### Explanation for the choice of comparators {6b}

Appropriate diabetes management is essential to reduce diabetes-related morbidity and mortality among people living with diabetes. Diabetes management can include monitoring blood glucose, medication and appointment adherence, physical activity, healthy nutrition, and annual screenings which can include blood work, eye exams, and sensation testing of the extremities [21]. Being able to achieve proper diabetes management relies on both patient and provider factors. Patients may need social support services, access to health care services, and culturally and linguistically appropriate resources. Providers’ beliefs and attitudes of the disease, ongoing interactions and relationship between patient and provider, and internal environment of their health system can also contribute to patients’ diabetes self-management [22–24]. Given these factors, we selected three interventions for supporting diabetes management. First, DSMT, also known as diabetes self-management education and support (DSMES), is an education program for people living with diabetes that employs an evidence-based curriculum and has led to improved diabetes-related health outcomes [25]. Second, RGM refers to an automated process of transmitting blood glucose levels directly from a smart phone to a healthcare provider. Limited research exists, but those that do, have found regular engagement with technology like RGM have led to improved health outcomes [13]. Finally, in the second rerandomization we will include tailored support from a CHW. CHWs are frontline public health workers who are trusted members of and/or have a particularly close relationship with the community of interest [26]. Evidence has shown that CHWs can be a cost-effective way to improve chronic disease management and address the social determinants of health that underlie health inequities [27, 28].

### Intervention description {11a}

In the first randomization, study participants will be randomly assigned to either the DSMT or RGM study arms after completion of the enrollment and baseline data collection. At 6 months, they will attend an in-person appointment for HbA1c lab work (if it has been more than 3 months since their last HbA1c lab work) or HbA1c point of care testing. Depending on their HbA1c, they will continue in the first randomization arm, or they will be rerandomized. Each intervention is described below.

#### Diabetes self-management training

Participants who are randomized to a DSMT arm will be part of a small-group four-part diabetes education series. Groups will consist of 5–8 participants and will be led by a diabetes educator. Each session will take place in person, will be approximately 2 h in length, and will be offered at the same day and time, every other week. Cohorts will move together through the series. Classes will include (1) Overview and monitoring of diabetes, (2) Healthy eating and exercise, (3) Importance of diabetic medication, and (4) Living with diabetes. If participants are unable to attend a class, they will be offered a makeup class by joining the missed session of another cohort.

#### Remote glucose monitoring

Participants who are randomized to the RGM arm will work with a study team member to download and set up a profile with the Glooko application on their personal smartphone device at the time of enrollment. Participants will receive a 1-h education session, where a study team member will assist with downloading the Glooko application, sync their Glooko-compatible meter, insulin pump, or continuous monitoring device, and provide education on Glooko’s features. After the education session, the participant will be instructed to use the application over the next 6 months. Participant glucose levels will be automatically transmitted to the electronic medical record (EMR), where providers can monitor the data remotely. The participant will also be able to track their weight, blood pressure, exercise, log food and meals, set reminders, and make notes on the application. Participants are provided with the research team’s contact information in case technical support is needed. The research team will also reach out to each participant every 2 months to provide technical assistance and encourage engagement with the application.

#### Community health worker support

CHWs will be introduced 6 months into the trial at rerandomization for participants whose HbA1c has not improved by at least one percentage point (Fig. 2. IDEA Study SMART Design). All patients paired with a CHW will be screened for social needs utilizing a standard social determinants of health (SDoH) screening tool, will receive referrals to resources to address unmet social/health needs, and will be actively navigated to health and social service resources as appropriate.

For participants originally randomized to the RGM intervention, the CHWs will focus on enhancing uptake and use of the Glooko application. During the initial in-person visit with the CHW, which will take place either at the clinic or in the participant’s home, the CHW will screen for SDoH needs, provide referrals to address unmet needs, and assess participant barriers to regularly measuring and/or reporting glucose levels using Glooko (e.g., connection issues between device and phone, barriers to transmitting information to provider). The CHW will also support the patient in overcoming barriers through individualized education, motivational interviewing, goal-setting, and trouble-shooting technological and resource challenges. Following the initial visit, the CHW will follow up with the patient at least monthly by phone for 6 months to assess adherence with RGM, trouble shoot additional barriers, and reinforce education concerning the Glooko application. The CHW will also provide active support to the participant in connecting with resources to support health and social needs. Additional in-person visits will occur during the time interval as needed.

For participants originally randomized to the DSMT study arm, the CHW will focus on reinforcing key messages presented during DSMT sessions. During the initial in-person visit with the CHW, which will take place either at the clinic or in the participant’s home, the CHW will screen for SDoH needs, provide referrals to address unmet needs, and assess knowledge gained during the group DSMT sessions, self-efficacy to manage diabetes, and application of learnings. The CHW will support the patient in overcoming deficits in knowledge and barriers in application of knowledge via additional customized, one-on-one education sessions, motivational interviewing, goal-setting, and navigation support. Following the initial visit, the CHW will follow-up with the patient at least monthly by phone, scheduling additional in-person visits as needed for up to 6 months. Additional in-person visits will occur during the time interval as needed.

### Criteria for discontinuing or modifying allocated interventions {11b}

This study is designed to cause minimal harm to the research participants while producing the most benefit. Topics discussing with the diabetes educator and CHW may be related to sensitive topics such as their current or past medical conditions, including those related to diabetes and weight, specific laboratory results and lifestyle choices such as eating habits and physical activity. All staff are trained to identify and support participants who may be uncomfortable. At the beginning of each DSMT sessions, the diabetes educator will inform participants that they are free to leave at any time if they are uncomfortable. This will not jeopardize their participation in the study nor will it impact their relationship with Sinai Chicago as a patient. All adverse reactions will be reported to the patient’s primary clinical team and the IRB. One of the PIs (JJ) leads weekly meetings with the implementation team which will be the first-line opportunity to identify and discuss any concerns across the participant. The PIs meeting biweekly to discuss updates and challenges, and the Subject Matter Expert (SME) Committee, described in more detail below, consists of a team of diabetes and community health specialist who will review cases as needed to determine any necessary modifications.

### Strategies to improve adherence to interventions {11c}

Participants randomized to the DSMT study arm will receive a call or text message from the diabetes educator prior to each session to remind them of the class. For participants who are not able to join one of their regularly scheduled sessions, a make-up class will be offered to maximize adherence. Participants randomized to the RGM study arm will receive a call or text message from a research staff member every 2 months to provide technical assistance and encourage engagement with the application. Finally, participants randomized to the CHW study arm will receive a call or text message from the CHW prior to each one-on-one visit. We will measure intervention adherence using attendance (number of DSMT sessions attended), RGM utilization (number of blood glucose readings uploaded to Glooko and number of syncs to Sinai’s medical record system), and adherence with CHW enhancements: (1) completing an initial education session, (2) completing a SDoH screener, (3) connecting with the CHW via phone or in person at least three times over 6 months.

### Relevant concomitant care permitted or prohibited during the trial {11d}

All study participants will be able to seek standard of care for diabetes management during the study period. To prevent contamination, individuals who are randomly assigned to the DSMT study arm will not be provided with a Glooko license until the study is complete. Individuals in the RGM study arm will not be offered specialized diabetes education from a diabetes educator until the study is complete. Individuals will only be offered resources as it pertains to the standard of care.

### Provisions for post-trial care {30}

Upon completion of the trial, all study participants will be offered a Glooko license for RGM utilization to support ongoing monitoring of their blood glucose levels. Individuals will also be provided with diabetes education as necessary and as covered by their insurance. The Centers for Medicare and Medicaid cover up to 10 h of diabetes education sessions per year. As needed, patients will also be offered an appointment with a CHW employed by the Center to support any social needs.

### Outcomes {12}

The primary outcome of interest is HbA1c, a clinical measure of diabetes management. We will measure the change in HbA1c from baseline (must be from within the 90 days prior to enrollment) to the 12-month study end point. HbA1c will be measured every 6 months, with the first 6-month measurement serving as our tailoring variable for the SMART. Patients with an HbA1c reduction of at least one percentage point will be considered responders [29].

The secondary outcomes of interest are implementation outcomes and patient-centeredness. The implementation outcomes that will be measured include appropriateness, fidelity, sustainability, and patient-centeredness. We define appropriateness as the perceived fit or compatibility with the patient population, staff, and organization; fidelity as the degree to which the intervention is implemented as intended; and sustainability as the extent to which the treatment is sustained or maintained. Patient-centeredness will be measured using the six dimensions defined by the Institute of Medicine. See Table 2 for a full list of outcomes measures and data sources.

**Table 2:** Outcomes and data sources

**Domain**	**Specific measurement**	**Specific metric**	**Method of aggregation**	**Timepoint**	**Data source**
Clinical Effectiveness	HbA1c	Change from baseline	Proportion of participants with a decrease of at least 1 percentage point	6 and 12 months post enrollment	EMR
Fidelity	Active RGM Usage	Value at intervention completion	Binary value (Y/N: at least one upload of data within a 30-day period and at least 16 blood glucose entries within a 30-day period)	6 months after enrollment or rerandomization^a^	Glooko Reports
	DSMT attendance	Value at intervention completion	Binary value (Y/N: attended at least three of four sessions)	6 months after enrollment or rerandomization^a^	DSMT Attendance Sheets
	Adherence to CHW engagement	Value at intervention completion	Binary value (Y/N: completed initial education session, SDoH screener, and at least three meetings with CHW)	6 months after enrollment or rerandomization^a^	CHW Log Sheet
Appropriateness	Perceived appropriateness, fit, and compatibility with patient population and organization from the perspectives of Center staff and patients	Qualitative self-reported experiences	N/A	Between 3 and 6 months after enrollment or rerandomization^a^	Center Staff Interviews Patient Focus Group Discussions
Sustainability	Short Form-Diabetes Empowerment Scale (SF-DES)	Change from baseline	Mean	6 and 12 months post enrollment	RedCAP Study Database
	Problem Areas in Diabetes (PAID) Survey	Change from baseline	Mean	6 and 12 months post enrollment	RedCAP Study Database
Patient-Centeredness	Respect for patients’ values, preferences, and expressed needs Coordinated and integrated Provide Information, communication, and education Ensure physical comfort Provide emotional support Involve family and friends	Qualitative self-reported experiences	N/A	Between 3 and 6 months after enrollment or rerandomization^a^	Patient Focus Group Discussions
Covariates	Race/ethnicity, Age, Sex, Zip code, Insurance status	Value	Distribution across categories	Baseline	EMR RedCAP Study Database

*RGM* remote glucose monitoring, *DSMT* diabetes self-management training, *CHW* community health worker, *EMR* electronic medical record, *SDOH* social determinants of health, *N/A* not applicable, qualitative measure

^a^Depending on initial and/or secondary if participant was assigned to RGM at their initial or rerandomization point

### Participant timeline {13}

**Fig. 3:**
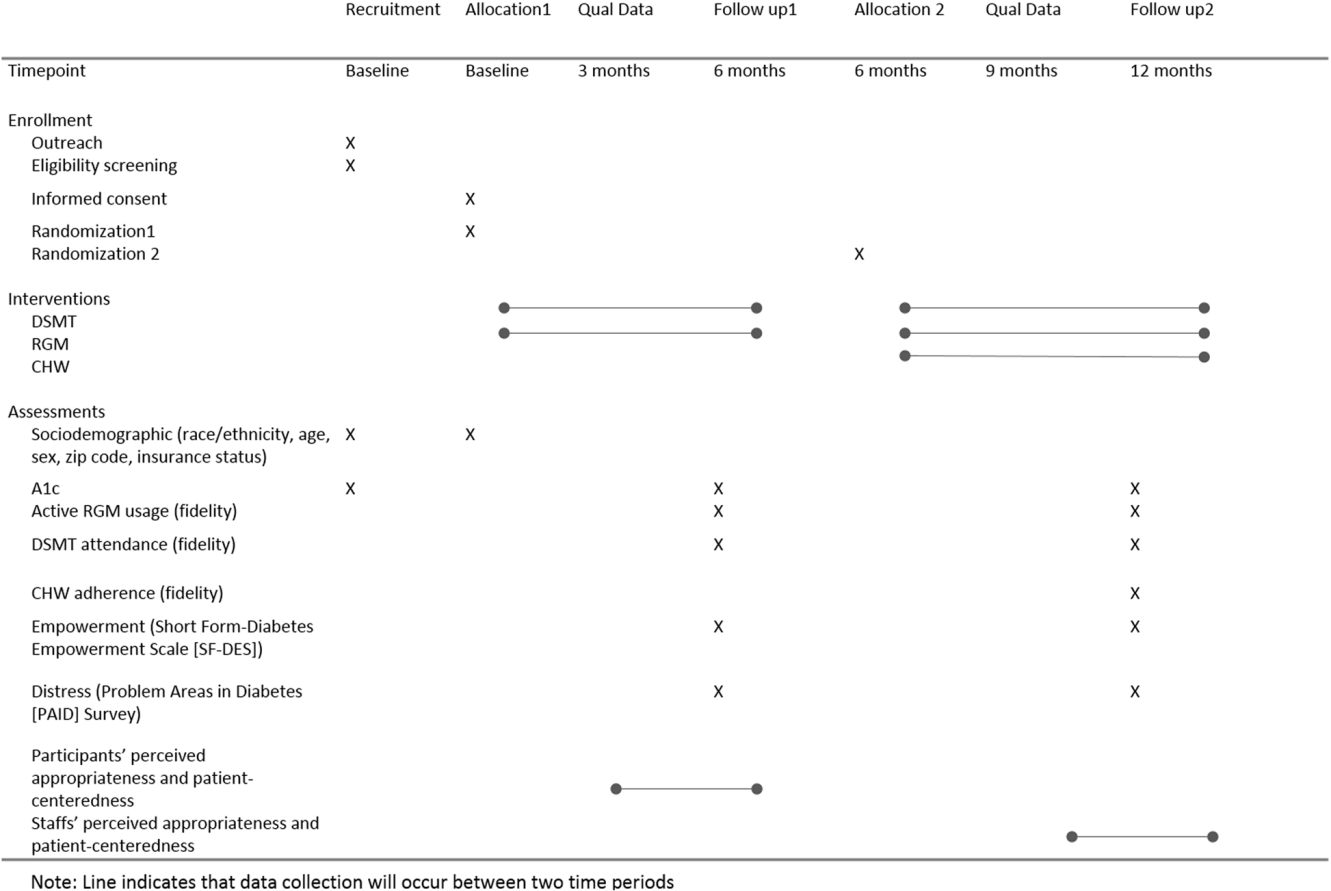
Schedule of enrollment, interventions, and assessments

See Fig. 3*. Schedule of enrollment, interventions, and assessments* for the full participant timeline.

### Sample size {14}

The sample size calculation, performed using G*Power (version 3.1), is based on the primary outcome of HbA1c over the 12-month study period, with the primary predictor being the six treatment conditions taken over the course of the study. The Null (H0) and Alternative (H1) Hypotheses are H0: There is no difference in the mean change in HbA1c among the six treatment groups, and H1: There is a significant difference in the mean change in HbA1c among the six treatment groups. The analysis assumes a significance level of 5%, 80% power, six treatment groups, equal group allocation, and a medium effect size of *f* = 0.25. The effect size of *f* = 0.25 was selected based on Cohen’s conventions for effect sizes in behavioral sciences, where 0.10 represents a small effect, 0.25 represents a medium effect, and 0.40 represents a large effect. A medium effect size is considered appropriate for this study, as it balances the detection of meaningful clinical differences with practical sample size considerations. Previous studies on diabetes management interventions such as DSMT and RGM have reported effect sizes in this range, indicating that a medium effect size is a reasonable expectation for interventions aimed at reducing HbA1c [13, 25].

The sample size estimation properly accounted for the multiple groups and comparisons inherent in the SMART design. Specifically, we conducted the sample size estimation for detecting a difference in HbA1c change across the six treatment groups, using an ANOVA framework, which inherently accounts for multiple comparisons. To ensure robustness and accommodate potential dropouts, we inflated the sample size by 20%. Thus, the minimum required sample size is 216 patients, and to account for an anticipated dropout rate, we aim to recruit 270 patients. This approach ensures that the study is adequately powered to detect significant differences across all treatment conditions, providing a solid basis for our subsequent analyses.

### Recruitment {15}

Potential SMART participants are screened using a two-step process. First, a weekly patient list is extracted from the EMR, cleaned in excel, and imported into the study’s REDCap database. A member of the study team will review each patient’s REDCap record and assess preliminary eligibility based on exclusionary medical conditions (see above for full list of exclusion criteria), living status, and the date and value of the most recent HbAlc level (within 90 days). Second, participants that may be eligible based on a preliminary assessment of their medical record will be contacted to complete a phone screening. During the phone screening, a member of the study team will obtain patient-reported eligibility information including race/ethnicity, confirmation of medical conditions, smartphone access, and ability to attend in-person diabetes education sessions. Participants who are eligible are invited to attend an in-person enrollment appointment. Participants who may be eligible, but do not have an HbA1c measurement within the past 90 days, will be encouraged to make an appointment with their provider to determine eligibility.

Focus group recruitment will utilize criterion sampling, using participation in the SMART study as the criterion for focus group eligibility. A total of 12 focus groups will be conducted over the course of the project. Three months after the initial SMART study recruitment, patients within each of the two initial study conditions will be randomly ordered, and a member of the study team will go down the list of randomized patients and contact each patient by phone to describe the focus group, timeline, and benefits and risks of participation. This will continue until eight patients per study condition agree to participate. Participants will be scheduled to participate in a 2-h focus group. The same recruitment strategy will be used for the final eight focus groups after rerandomization.

Center staff (e.g., clinicians, diabetes educators, community health workers) actively engaged in delivery of one of the three interventions will be invited to participant in an interview. We will seek role variability with at least one staff member from each type of role (*n* = 10). After contacting the staff member by email and confirming their interest in participating, a study team member will provide the staff person an in-depth description of the study, the timeline, and benefits and risks of participation. Eligible Center staff will be scheduled to participate in a 45-min semi-structured interview.

## Assignment of interventions: allocation

### Sequence generation {16a}

The sequence of study arm assignment will be done using a modified version of the sequentially numbered, opaque sealed envelopes (SNOSE) [30]. In order to empower study participants to ensure they feel like part of the study and have a sense of autonomy, we created randomization envelopes that participants will be able to choose from. Following guidance from Doig & Simpson, we took several steps to design and create randomization allocation envelopes that will be concealed from the participant and the researcher. We developed a detailed operating procedure and provided this to a staff member who is unfamiliar with the research study. For the first randomization, the staff member printed out 150 pieces of paper that say “DSMT” and 150 pieces of paper that say “RGM”. Starting with the “RGM” paper, they folded each one and put it into a completely opaque security envelope. Each envelope was sealed and the staff member wrote their initials, in pen, on the top of the envelope seal. The same steps were repeated for the DSMT paper. Once all 300 envelopes were created, the staff member created two boxes of envelopes, stratified by recruitment site. For the first box, for patients recruited from Mount Sinai Hospital, the staff member selected 75 RGM envelopes and 75 DSMT envelopes. These were shuffled thoroughly and placed in the box marked “MSH”. For the second box, for patients recruited from Holy Cross Hospital, the staff member shuffled the remaining 75 RGM envelopes and 75 DSMT envelopes and placed them in the box marked “HCH”. These boxes and their sealed envelopes were placed in a study team member’s locked office to prepare for recruitment.

Randomization envelopes were created for the second round of randomization at 6 months following a similar protocol. In this case, the staff member printed 60 pieces of paper that say “CHW,” 60 pieces of paper that say “RGM,” 60 pieces of paper that said DSMT, and 60 pieces of paper that say “CHW + RGM.” We assumed that a certain number of participants would improve by at least one percentage point and would not be randomized. After creating the 240 envelopes, the staff member created two boxes of envelopes, stratified by the initial treatment assignment. In the first box, for participants initially assigned to DSMT, the staff member shuffled and placed the CHW and RGM envelopes in the box marked “DSMT.” In the second box, for participants initially assigned to RGM, the staff member shuffled and placed the remaining DSMT and CHW + RGM envelopes in the box marked “RGM.”

### Concealment mechanism {16b}

Once the participant completes the intake and randomization, the research *staff member* will assign a participant identification number to the participant. The first number is based on the recruitment site (1 = Mount Sinai Hospital; 2 = Holy Cross Hospital). The second and third identification digits are based on the initial study arm (77 = RGM group; 88 = DSMT group). The last three digits are based on the order in which they enroll in the study, consecutively beginning with 001. This identification number will be documented on the intake form and in REDCap.

### Implementation {16c}

The two research staff involved in the recruitment will have no involvement in the allocation of participants to the interventions. The participant will select an envelope at random, and once selected, that will be the final envelope. The research staff member will document the assignment in REDCap.

## Assignment of interventions: blinding

### Who will be blinded {17a}

Only the statistician will be blinded to the intervention assignments. They will receive a de-identified dataset that will list a unique identifier other than the one noted above since the identifier above includes information related to the study arm. The dataset will include a value indicating which study arm(s) participants have been assigned to at each randomization point, but the statistician will be unaware of how the code corresponds to study arms.

### Procedure for unblinding if needed {17b}

The participants and research team located at Sinai Chicago will not be blinded to the study conditions. Only the statistician, an independent consultant, will be blinded to the study conditions. The research team at Sinai Chicago will have a matrix that links participant identifiers to the codes provided to the statistician.

## Data collection and management

### Plans for assessment and collection of outcomes {18a}

#### Intervention outcome measure

The primary outcome of our study is change in HbA1c which will be calculated as three measures. We will collect HbA1c at baseline (no more than 90 days prior to patient intake), 6-month follow-up, and 12-month study end point. First, we will use change in HbA1c as the tailoring variable for our SMART. We will calculate change in HbA1c from baseline intake to 6-month follow-up. Then we will calculate change in HbA1c from baseline to 12-month follow-up and from 6-month to 12-month follow-up. Because HbA1c is an average measure of blood sugar levels of the past 3 months, clinicians often order an HbA1c test every 90 days. If it has not been at least 90 days since the patient’s last HbA1c at the 6- or 12-month follow-up, we will use an HbA1c point of care test kit (HbA1c Now by Bayer) to measure the patient’s HbA1c.

The secondary outcomes are implementation and service outcomes: fidelity, appropriateness, sustainability, and patient-centeredness. Fidelity will be measured by intervention adherence. Active RGM adherence will be measured using monthly data reports from Glooko. These will be provided at the patient level and will include number of syncs to Sinai Chicago’s EMR per month and number of blood glucose entries uploaded to Glooko per month. DSMT adherence will be calculated using attendance sheets. Participants will be adherent if they attend at least three of the four sessions. Make-up sessions will be made available to participants. Finally, adherence to the CHW intervention will include three activities: (1) completing an initial education session, (2) completing an SDoH screener, and (3) completing at least three in-person or phone touchpoints. These will be documented in the CHW tracking log and uploaded into REDCap. Appropriateness will be gathered from patient focus groups and staff interviews. Sustainability of diabetes-related learnings and self-efficacy will be measured by two validated scales: the short-form diabetes-related empowerment scale (DES-SF) and the Problem Areas in Diabetes (PAID) scale [31, 32]. Appropriateness and patient-centeredness will be collected using qualitative data. Questions to assess appropriateness will include patient and staff perceptions of appropriateness, fit and compatibility with the patient population and organizations. Questions to assess perceived patient centeredness will be related to the Institute of Medicine’s six domains of patient centeredness: (1) Respect for patients’ values, preferences, and expressed needs; (2) Coordinated and integrated; (3) Provide Information, communication, and education; (4) Ensure physical comfort; (5) Provide emotional support; (6) Involve family and friends.

### Plans to promote participant retention and complete follow-up {18b}

To minimize attrition, the research staff will call or send a text message to all study participants at least every 2 months. Those randomized to an RGM arm will receive communication from a research team member every 2 months to provide technical assistance as needed for the Glooko application. Those randomized to a DSMT arm will receive a call or text message reminder from the diabetes educator prior to each DSMT session. If a participant is unable to attend a session, the diabetes educator will offer a make-up session based on the participant’s availability. During the initial enrollment appointment, the research team will confirm or collect extensive contact information including mailing address, home and cell phone number(s), e-mail addresses, and contact information for up to three additional individuals.

### Data management {19}

Study data will be collected and managed using REDCap electronic data capture tools hosted at Sinai Chicago [33, 34]. REDCap (Research Electronic Data Capture) is a secure, web-based software platform designed to support data capture for research studies, providing (1) an intuitive interface for validated data capture; (2) audit trails for tracking data manipulation and export procedures; (3) automated export procedures for seamless data downloads to common statistical packages; and (4) procedures for data integration and interoperability with external sources. Participant data will be entered directly into the study’s REDCap database by a study team member or collected on paper and entered into REDCap by a study team member within one business day. Only study team members will have access to the REDCap database. Randomly selected data collected on paper and later entered into REDCap will be periodically reviewed to ensure data quality. Paper copies of data collection forms, signed informed consent forms, and signed HIPAA authorization forms will be stored in a locked drawer at the study site. Any exports of study data from REDCap, audio recordings, notes (Word documents) and transcripts (Word documents), will all be saved on a secure study site server and only study team members will have access to the folder storing all materials. All paper files will be stored until 5 years after study completion and will then be destroyed. Electronic data will be destroyed or fully de-identified 5 years after study completion.

### Confidentiality {27}

Procedures are in place to ensure confidentiality and provide full informed consent. The consent form and information provided to the participant will have a contact phone number to call if they have questions or concerns about any aspect of the study. The following policies and procedures will be adopted to maintain privacy and confidentiality:

1. Data will be collected and analyzed by research staff only, password protected and housed in a secure folder on a secure server, or in a secure REDCap database, only accessible to study team members.
2. All study staff will receive training on maintaining the privacy and confidentiality of individual information, including HIPAA training.
3. The participants’ records will be maintained in a password-protected project database until 5 years after study completion and will then be destroyed or fully de-identified.

All paper study files, including data collection forms, signed consent forms, and signed HIPAA forms will be stored until 5 years after study completion and will then be destroyed. Electronic data files will be destroyed or fully de-identified 5 years after study completion.

### Plans for collection, laboratory evaluation, and storage of biological specimens for genetic or molecular analysis in this trial/future use {33}

We will not be collecting or storing biological specimens for future use. All lab information comes from HbA1c results ordered by a provider for standard of care. During the point of care testing, no biological specimens are being stored. Once the point of care testing is complete and resulted, information will be documented in the medical record and the test will be discarded.

## Statistical methods

### Statistical methods for primary and secondary outcomes {20a}

In our quantitative analysis, we will employ descriptive statistics to provide a comprehensive overview of our data, calculating frequencies and percentages for categorical variables, and means with standard deviations or medians with interquartile ranges for continuous variables, paying special attention to any outliers.

#### Primary outcomes

To optimize the adaptive intervention and understand the causal effects of each proposed sequence, we will employ a SMART design. This design is particularly advantageous for chronic diseases like diabetes, where there is significant heterogeneity in response to treatment. The SMART design allows for re-randomization based on a tailoring variable, which in our study is the change in HbA1c at 6 months, enabling patients to receive an adaptive intervention that is most suited to their needs. To address potential imbalances in randomization, we will use stratified and block randomization techniques.

For primary analysis, we will employ linear regression models adjusting for baseline characteristics utilizing type III sum of squares to account for these discrepancies. Sensitivity analyses will include inverse probability weighting to account for any imbalances. To assess the overall HbA1c reduction by treatment condition and the average patient percentage point reduction in HbA1c from baseline to 12 months within each of the six treatment conditions, we will utilize one-way ANOVA for mean comparisons, followed by pairwise comparisons with a Tukey post hoc test to correct for multiple comparisons. In cases of non-normal data distribution, we will opt for Kruskall-Wallis tests and a Dunn post hoc test with Hommel multiple comparison *p*-value corrections.

Linear regression will be employed to adjust for additional covariates that may confound the relationship between treatment and outcome, such as baseline characteristics with residual confounding post-randomization, and baseline and 6-month HbA1c values. We will also test for possible interactions of covariates with the treatment variable, reporting model adjusted slopes (β_ADJ_), 95% confidence intervals, model diagnostics, and goodness of fit (via R^2^).

To determine the optimal dynamic treatment regime (DTR) that maximizes the greatest benefit of percentage reduction in HbA1c at each randomization stage of the study, we will employ a Q-learning algorithm. This algorithm utilizes a Q-function *Q* (s_*i*_, *t*_*i*_) to measure the quality of assigning a treatment (*t*_*i*_) to a patient at a specific stage (*s*_*i*_), with higher reductions in HbA1c percentages yielding higher quality. Regression models will be used to estimate these Q-functions, taking into account the outcome, treatment types, and all other covariates of interest. We will assess model diagnostics and fit, and test interactions to retain significant results. With the Q-function known, the optimal DTR π^∗^(*s*_*i*_, *t*_*i*_) is determined by the treatment (*t*_*i*_) which maximizes *Q* (*s*_*i*_, *t*_*i*_) given the stage (*s*_*i*_).

#### Secondary outcomes

For secondary outcomes, including implementation outcomes (fidelity, appropriateness, sustainability) and patient-centeredness, we will use a combination of qualitative and quantitative methods. Descriptive statistics will summarize categorical variables as frequencies and percentages and continuous variables as means with standard deviations or medians with interquartile ranges. Fidelity to the interventions will be assessed by measuring RGM adherence (data uploads and blood glucose entries in Glooko), DSMT attendance rates, and CHW engagement (completion rates of initial education sessions, SDoH screeners, and follow-up meetings). Logistic regression will identify predictors of adherence.

Appropriateness and sustainability will be evaluated through qualitative analysis of focus group and interview data, which will be transcribed, coded, and analyzed thematically. Quantitative analyses will compare scores from validated surveys (Short Form-Diabetes Empowerment Scale (SF-DES) and Problem Areas in Diabetes (PAID)) between groups using paired *t*-tests or Wilcoxon signed-rank tests for within-group comparisons and independent *t*-tests or Mann–Whitney *U* tests for between-group comparisons.

In our secondary analysis, patients in treatment scenarios D or F will complete the DES-SF and PAID at 6 and 12 months, with scores compared between treatment scenario groups using a paired *t*-test or paired Wilcoxon test. For exploratory analysis, we will follow the same process, though our focus will be on assessing trends in both primary and secondary outcomes due to potential limitations in statistical power from the addition of the CHW enhancement.

### Interim analyses {21b}

In the context of the current study, which primarily aims to evaluate the effectiveness of different sequences of interventions to reduce HbA1c levels, the necessity of conducting interim analyses is recognized. These analyses are crucial for ensuring the quality and consistency of our data, as well as for assessing the fidelity and appropriateness of the interventions being implemented.

At the 6-month mark of our study, we plan to conduct an interim analysis. The objectives of this analysis are multifaceted. Firstly, we aim to assess the quality and consistency of the data being collected across different sites and interventions, ensuring that any discrepancies or issues are identified and addressed promptly. Secondly, we intend to gain an early understanding of the fidelity of the interventions, including DSMT attendance, RGM uptake and adherence, and CHW support. Additionally, we will evaluate the appropriateness of the interventions from the perspectives of both providers and patients, ensuring that the interventions are well-received and fit the needs of the target population.

The interim analysis will be conducted using predefined decision rules to ensure rigor and minimize bias. For data quality and consistency, we will review completeness, accuracy, and consistency of the collected data. Any discrepancies will be addressed by revising data collection procedures and providing additional training to data collectors if necessary. To evaluate fidelity, we will calculate adherence rates to each intervention component. If fidelity rates fall below predefined thresholds (e.g., < 75% attendance for DSMT, < 50% RGM data uploads), we will implement corrective actions such as protocol modifications or enhanced participant engagement strategies.

The process of conducting the interim analysis is designed to be rigorous and unbiased. This will be achieved through several measures. Firstly, the interim analysis will be conducted by a subgroup of the research team that includes experts in biostatistics who are not involved in the day-to-day data collection or intervention delivery, ensuring that the analysis is conducted independently and objectively. To prevent bias, the data analysts will be blinded to the specific intervention groups when feasible, helping to ensure that the analysis and interpretation of the data are not influenced by preconceived expectations about the outcomes of the interventions. Additionally, all data collection and analysis procedures will follow standardized protocols to ensure consistency and reliability, with any deviations from the protocol being documented and addressed systematically. The criteria for making any modifications to the study based on interim analysis findings will be predefined, including specific thresholds for data quality, fidelity, and appropriateness metrics that will trigger corrective actions. By adhering to these predefined rules, we minimize the risk of bias that could arise from ad hoc decision-making. Finally, all steps of the interim analysis, including the decision-making process and any modifications implemented, will be thoroughly documented to ensure transparency.

### Methods for additional analyses (e.g., subgroup analyses) {20b}

In our additional analyses, we aim to explore the potential moderating effects of various demographic and socioeconomic factors on the effectiveness of our stepped-care TBC intervention. Specifically, we will conduct subgroup analyses to investigate whether the impact of the intervention varies across different levels of Race/ethnicity, Age, Sex, and Insurance status. We will employ interaction terms in our regression models to test for statistical interactions between the treatment conditions and these covariates. This will help us to identify any differential effects of the intervention across various subgroups, providing insights into the equity of our intervention and highlighting any potential disparities in its effectiveness. For instance, we will include interaction terms between the treatment conditions and Race/ethnicity to assess whether the intervention’s impact on HbA1c reduction differs between Black/Latine patients and other racial/ethnic groups. Similarly, we will investigate potential age and sex interactions to explore whether the intervention is more or less effective in older versus younger patients, and in male versus female patients.

### Methods in analysis to handle protocol non-adherence and any statistical methods to handle missing data {20c}

In order to address potential issues related to protocol non-adherence in our SMART design, we will employ specialized analytical methods that are tailored to adaptive interventions. Unlike traditional clinical trials where intention-to-treat (ITT) and per-protocol (PP) analyses are commonly used, our study’s design necessitates a different approach due to the planned adaptability of the interventions based on participants’ responses.

We will utilize Q-learning and g-estimation strategies to analyze the primary and secondary outcomes. These methods are specifically designed for adaptive interventions and will allow us to estimate the causal effects of the different sequences of treatments while accounting for the adaptive nature of the intervention. By employing these methods, we aim to obtain unbiased estimates of treatment effects, even in the presence of protocol non-adherence due to the adaptive design.

To handle missing data, we will implement multiple imputation (MI) techniques using a fully conditional specification (FCS) approach, also known as multiple imputation by chained equations (MICE). The imputation model will include variables predictive of the missing values and outcomes, such as baseline HbA1c, age, sex, race/ethnicity, intervention group, and socioeconomic status. Imputation will be performed separately within each treatment arm to account for differences in data distributions, generating 20 imputed datasets to ensure stability and reliability. Each imputed dataset will be analyzed using the specified statistical models for primary and secondary outcomes, including one-way ANOVA, linear regression models, and Q-learning algorithms. The results from the imputed datasets will be combined using Rubin’s rules to produce valid statistical inferences accounting for the uncertainty due to missing data.

To validate the imputation model, we will compare the distribution of imputed values with observed values. Sensitivity analyses will be conducted to assess the robustness of our findings under different missing data mechanisms, comparing results from multiple imputation with those obtained from complete-case analyses and other missing data approaches. This approach aims to ensure accurate and reliable estimates, maintaining the validity of our study’s findings.

### Plans to give access to the full protocol, participant-level data, and statistical code {31c}

We will follow the U.S. Department of Health and Human Services’ guidance on dissemination of trial information. The current study is registered under clinicaltrials.gov for public viewing and results. Results will include the following: demographic information, baseline characteristics, outcomes, analyses, adverse events, and an analysis plan. Results will be submitted no later than 1 year after the completion of final data collection. We will provide study protocol, statistical analysis plan, and the clinical study report. Ms. Jacobs will oversee all data storage procedures, including maintenance of security and confidentiality. Data will be gathered electronically in REDCap or will be captured on paper and entered into REDCap within 48 business hours. Because our statistician is a consultant, we have a data sharing agreement and business associate agreement in place. Although we have these agreements in place, we will strip all data of identifiable information before electronically sharing this information.

## Qualitative analysis methods

Qualitative analysis of focus group and interview data will be ongoing as data is collected. Interviews with staff and focus groups with patients will be recorded, transcribed verbatim and analyzed using a thematic approach in qualitative analysis software. An abductive coding approach will be used, beginning with a priori codes based on the frameworks used to develop interview and focus group guides, while also allowing for codes to emerge through analysis for a deeper understanding of the relationship between concepts [35]. Two study team members trained in qualitative data analysis will independently code transcripts, reviewing each other’s work and discussing discrepancies to arrive at consensus. To ensure reliability, a minimum of two coders will analyze the data, with 25% of transcripts randomly selected for independent double-coding [36].

## Oversight and monitoring

### Composition of the coordinating center and trial steering committee {5d}

This study is being conducted by Sinai Chicago with partnership between a research institution, Sinai Urban Health Institute, and a clinical entity, the Sinai Center for Diabetes and Endocrinology. The study will also be guided by two committees: a Patient Advisory Committee (PAC) and a Subject Matter Expert (SME) Committee. The PAC was formed in 2021 based on a previous study. It is comprised of seven individuals who are adults, 18 years of age or older, living with diabetes, many of which are current patients of Sinai Chicago. The PAC will meet quarterly to advise on recruitment, participant engagement, and interpretation of findings. The SME Committee is comprised of six individuals who will also meet quarterly. The purpose of this group is to advise on clinical or programmatic issues that may arise related to a patient’s care or medical situation. The SME consists of an endocrinologist, the practice manager, a nurse diabetes educator, and a community health worker.

### Composition of the data monitoring committee, its role and reporting structure {21a}

According to the 21 Code of Federal Regulations (21 CFR 312.50, 312.56, 812.40, and 812.46), a data monitoring committee is necessary for studies evaluating new drugs, biologics, or devices [37]. Because this study is not evaluating a new drug, biologic, or device, a data monitoring committee is not necessary.

### Adverse event reporting and harms {22}

The current study presents minimal risk to participants. However, potentially uncomfortable discussions may occur as it relates to participants’ previous or current medical history. All research and program staff involved in the study are responsible for reporting adverse events, including any unanticipated harms to participants, to the study PIs. We will follow the Office for Human Research Protections (OHRP) guidance for “reviewing and reporting unanticipated problems involving risks to subjects or others and adverse events” to classify adverse events as unexpected adverse events and/or serious adverse events, when applicable [38]. All adverse events will be documented in REDCap within two business days, using a detailed form specifically created to document adverse events. The form will be reviewed within two business days of the initial documentation. All documented adverse events will automatically be reported to study PIs. If an adverse event is determined to be a serious adverse event, it will be reported to the IRB as well.

### Frequency and plans for auditing trial conduct {23}

The implementation team, comprised of one PI (JJ), the senior evaluator, two research specialists, the community health worker, and the diabetes educator, meet weekly to review recruitment and enrollment data. The team members responsible for recruitment and enrollment bring any points of discussion to the group. The three PIs and the senior evaluator meet bi-weekly to discuss recruitment, enrollment, and any implementation or clinical challenges as recruitment progresses.

### Plans for communicating important protocol amendments to relevant parties (e.g., trial participants, ethical committees) {25}

Any changes to the study protocol will be submitted to the Mount Sinai Hospital Institutional Review Board (IRB) and will not be implemented until IRB approval. All investigators and study team members will be made aware of changes prior to submission and will be alerted immediately upon approval. Per IRB requirements, current participants will be alerted to protocol changes should it affect their participation. Contact information for all participants are kept secure within the study database.

### Dissemination plans {31a}

Study findings have the potential to inform optimization of evidence-based approaches to address diabetes and will be shared broadly. Researchers, practitioners, policymakers, and community partners are our key target audience. Findings will be disseminated through traditional academic pathways (e.g., presentations at national and local conference, peer-reviewed journal publications). We will also utilize the Dissemination Planning Tool, developed by Carpenter et al., to ensure our research findings are disseminated in a way that will broadly reach our target audience [39]. Unlike more academic methods of dissemination, we seek to communicate findings to diverse interested parties. We will use the Dissemination Planning Tool framework and our PAC to identify what we will disseminate, to whom, how we can extend our reach, how we will convey findings and results, how to evaluate our dissemination plan, and a timeline for dissemination.

## Discussion

Diabetes affects millions of Americans each year, including a disproportionate number of racial and ethnic minorities. It is increasingly expensive to our healthcare system and to individuals, and can have severe long-term health implications. Team-based care, and specifically interventions like diabetes education, leveraging technology like RGM, and integrating non-clinical staff like CHWs, have shown promise in improving health outcomes among certain populations [40, 41]. Yet there are still gaps in our understanding of how appropriate these interventions are for the population of interest, the degree to which we can maintain fidelity, and ultimately the potential change in HbA1c among specific populations. Specifically, various social determinants of health needs, which are linked to health outcomes, may not be addressed in the traditional patient-provider relationship. The intervention components of this study provide support towards better diabetes management will also addressing SDoH needs. Findings from this study will provide a nuanced perspective of the combination and sequence of interventions that best address the needs of specific patients.

We have identified some issues when developing the protocol that are important to document for future investigators, specifically those utilizing the SMART design. First, while random assignment using software, such as REDCap, is common in the trial literature, we chose to use SNOSE and allow research participants to make the envelope selection. Our randomization process, based on the work of other researchers [30, 42], allows us to effectively randomize participants, while also following equitable research principles that empower participants [43]. Second, to prevent contamination between study arms, we have identified steps to ensure the full clinical team at the Center is aware of patients that are participating in the study (e.g., information sheets provided to staff, documentation in the EMR), and asked that they not offer DSMT or RGM to currently enrolled participants. It has been particularly challenging to explain the extent to which education can still be provided to patients during standard clinical appointments. The distinction between DSMT and informal education provided during a standard care visit is nuanced. Finally, Sinai Chicago moved to a new EMR system in September 2023, right before recruitment began. Like any other organizational change, the team has required additional time to become familiar with the system, and work with information technology staff to create the correct reports.

This study uses an innovative trial design to measure the effectiveness of varying the sequence and combination of three interventions to support diabetes management among Black and Latine patients receiving care at a safety-net hospital. Guided by a health disparities framework, we will evaluate the appropriateness (Aim 1) and fidelity (Aim 2) of these interventions, compare the effectiveness of varying the sequence to reduce HBA1c (Aim 3), and measure the extent to which CHW support can reinforce DSMT and RGM uptake and adherence (Exploratory Aim 3a). By achieving these aims, we will build the evidence for optimizing equitable diabetes management and ultimately reducing racial and ethnic healthcare disparities for patients living in disinvested urban settings.

## Limitations

Study recruitment can be challenging with populations that have been historically harmed by medical and research institutions, leading to distrust of these institutions. To mitigate this challenge, we will rely on study team members and Center staff (including co-PI Dr. Wagener), who have relationships with patients, to support recruitment and to serve as champions for the study. We also acknowledge that retention may be a challenge as participants will be asked to continue with the study for 1 year. The research team has successfully navigated past retention challenges, specifically in studies of patients living with diabetes. Patients must have a smartphone to participate in the RGM intervention of the adaptive trial which could be a barrier for some patients.

## Trial status

Recruitment began on October 23, 2023, and is expected to conclude in September 2024. The current protocol is version 1.0, updated as of November 1, 2023.

### Supplementary Information

Supplementary Material 1.

## Data Availability

The study team will follow the U.S. Department of Health and Human Services’ guidance on dissemination of trial information. The current study is registered under clinicaltrials.gov for public viewing and results. The study protocol, statistical analysis plan, and clinical study report will be made available. Data collected as part of this study includes clinical data, qualitative recordings and transcripts, and quantitative surveys. All data will be stripped of identifiable information before being made available.

## Abbreviations

TBC: Team-based care
HbA1c: Hemoglobin A1c
DSMT: Diabetes self-management training
RGM: Remote glucose monitoring
CHW: Community health worker
SMART: Sequential multiple assignment randomized trial
SUHI: Sinai Urban Health Institute
MSH: Mount Sinai Hospital
HCH: Holy Cross Hospital
PI: Principal investigator
DSMES: Diabetes self-management education and support
EMR: Electronic medical record
SDoH: Social determinants of health
SF-DES: Short Form-Diabetes Empowerment Scale
PAID: Problem Areas in Diabetes Survey
SNOSE: Sequentially numbered, opaque sealed envelopes
REDCap: Research Electronic Data Capture
DTR: Dynamic treatment regime
ITT: Intention-to-treat
PP: Per protocol
PAC: Patient Advisory Committee
SME: Subject Matter Expert; IRB: Institutional Review Board
