# Supplementary material for: Improving Diabetes Equity and Advancing Care (IDEA) to optimize team-based care at a safety-net health system for Black and Latine patients living with diabetes: study protocol for a sequential, multiple assignment, randomized trial
Source: Trials. 2024 Jul 24;25:504. doi: 10.1186/s13063-024-08346-9 (PMC11270937; doi:10.1186/s13063-024-08346-9)
Supplement: Supplementary file 1 — Supplementary Material 1. [file 13063_2024_8346_MOESM1_ESM.docx]

ACE checklist for the main report

| Section/ Topic | Item No | Standard CONSORT 2010 checklist item | Extension for adaptive design randomised trials | Page No |
| --- | --- | --- | --- | --- |
| **Title and abstract** | 1a | Identification as a randomised trial in the title |  | 1 |
|  | 1b | Structured summary of trial design, methods, results, and conclusions (for specific guidance see CONSORT for abstracts)136137 | Structured summary of trial design, methods, results, and conclusions (for specific guidance see ACE for abstracts, table 3) | 1 |
| **Introduction** |  |  |  |  |
| Background and objectives | 2a | Scientific background and explanation of rationale |  | 2 |
|  | 2b | Specific objectives or hypotheses |  | 5 |
| **Methods** |  |  |  |  |
| Trial design | 3a | Description of trial design (such as parallel, factorial) including allocation ratio |  | 6 |
|  | 3b«^*^ |  | Type of adaptive design used, with details of the pre-planned trial adaptations and the statistical information informing the adaptations | 6 |
|  | 3c«3b^†^ | Important changes to methods after trial commencement (such as eligibility criteria), with reasons | Important changes to the design or methods after trial commencement (such as eligibility criteria) outside the scope of the pre-planned adaptive design features, with reasons | 13 |
| Participants | 4a | Eligibility criteria for participants |  | 8 |
|  | 4b | Settings and locations where the data were collected |  | 6 |
| Interventions | 5 | The interventions for each group with sufficient details to allow replication, including how and when they were actually administered |  | 9 |
| Outcomes | 6a^†^ | Completely defined pre-specified primary and secondary outcome measures, including how and when they were assessed | Completely define pre-specified primary and secondary outcome measures, including how and when they were assessed. Any other outcome measures used to inform pre-planned adaptations should be described with the rationale | 15 |
|  | 6b^†^ | Any changes to trial outcomes after the trial commenced, with reasons | Any unplanned changes to trial outcomes after the trial commenced, with reasons | 15 |
| Sample size and operating characteristics | 7a^†^ | How sample size was determined | How sample size and operating characteristics were determined | 16 |
|  | 7b^‡^ | When applicable, explanation of any interim analyses and stopping guidelines | Pre-planned interim decision-making criteria to guide the trial adaptation process; whether decision-making criteria were binding or non-binding; pre-planned and actual timing and frequency of interim data looks to inform trial adaptations | 25 |
| **Randomisation** | |  |  |  |
| Sequence generation | 8a | Method used to generate the random allocation sequence |  | 18 |
|  | 8b^†^ | Type of randomisation; details of any restriction (such as blocking and block size) | Type of randomisation; details of any restriction (such as blocking and block size); any changes to the allocation rule after trial adaptation decisions; any pre-planned allocation rule or algorithm to update randomisation with timing and frequency of updates | 18 |
| Allocation concealment mechanism | 9 | Mechanism used to implement the random allocation sequence (such as sequentially numbered containers), describing any steps taken to conceal the sequence until interventions were assigned |  | 19 |
| Implementation | 10 | Who generated the random allocation sequence, who enrolled participants, and who assigned participants to interventions |  | 19 |
| Blinding | 11a | If done, who was blinded after assignment to interventions (for example, participants, care providers, those assessing outcomes) and how |  | 20 |
|  | 11b | If relevant, description of the similarity of interventions |  | n/a |
|  | 11c^*^ |  | Measures to safeguard the confidentiality of interim information and minimise potential operational bias during the trial | 23 |
| Statistical methods | 12a^†^ | Statistical methods used to compare groups for primary and secondary outcomes | Statistical methods used to compare groups for primary and secondary outcomes, and any other outcomes used to make pre-planned adaptations | 24 |
|  | 12b«^*^ |  | For the implemented adaptive design features, statistical methods used to estimate treatment effects for key endpoints and to make inferences | 24 |
|  | 12c«12b | Methods for additional analyses, such as subgroup analyses and adjusted analyses |  | 26 |
| **Results** |  |  |  |  |
| Participant flow (a diagram is strongly recommended) | 13a^†^ | For each group, the numbers of participants who were randomly assigned, received intended treatment, and were analysed for the primary outcome | For each group, the numbers of participants who were randomly assigned, received intended treatment, and were analysed for the primary outcome and any other outcomes used to inform pre-planned adaptations, if applicable | n/a^1^ |
|  | 13b | For each group, losses and exclusions after randomisation, together with reasons |  | n/a^1^ |
| Recruitment and adaptations | 14a^†^ | Dates defining the periods of recruitment and follow-up | Dates defining the periods of recruitment and follow-up, for each group | 17 |
|  | 14b^§^ | Why the trial ended or was stopped | See expanded E&E text for clarification | n/a^1^ |
|  | 14c^*^ |  | Specify what trial adaptation decisions were made in light of the pre-planned decision-making criteria and observed accrued data | n/a^1^ |
| Baseline data | 15a«15^§^ | A table showing baseline demographic and clinical characteristics for each group | See expanded E&E text for clarification | n/a^1^ |
|  | 15b^*^ |  | Summary of data to enable the assessment of similarity in the trial population between interim stages | n/a^1^ |
| Numbers analysed | 16^§^ | For each group, number of participants (denominator) included in each analysis and whether the analysis was by original assigned groups | See expanded E&E text for clarification | n/a^1^ |
| Outcomes and estimation | 17a^§^ | For each primary and secondary outcome, results for each group, and the estimated effect size and its precision (such as 95% confidence interval) | See expanded E&E text for clarification | n/a^1^ |
|  | 17b | For binary outcomes, presentation of both absolute and relative effect sizes is recommended |  | n/a^1^ |
|  | 17c^*^ |  | Report interim results used to inform interim decision-making | n/a^1^ |
| Ancillary analyses | 18 | Results of any other analyses performed, including subgroup analyses and adjusted analyses, distinguishing pre-specified from exploratory |  | n/a^1^ |
| Harms | 19 | All important harms or unintended effects in each group (for specific guidance see CONSORT for harms)138 |  | n/a^1^ |
| **Discussion** |  |  |  |  |
| Limitations | 20^§^ | Trial limitations, addressing sources of potential bias, imprecision, and, if relevant, multiplicity of analyses | See expanded E&E text for clarification | 33 |
| Generalisability | 21^§^ | Generalisability (external validity, applicability) of the trial findings | See expanded E&E text for clarification | n/a^1^ |
| Interpretation | 22 | Interpretation consistent with results, balancing benefits and harms, and considering other relevant evidence |  | n/a^1^ |
| **Other information** | |  |  |  |
| Registration | 23 | Registration number and name of trial registry |  | 2 |
| Protocol | 24a«24 | Where the full trial protocol can be accessed |  | 2 |
| SAP and other relevant trial documents | 24b^*^ |  | Where the full statistical analysis plan and other relevant trial documents can be accessed | 2 |
| Funding | 25 | Sources of funding and other support (such as supply of drugs), role of funders |  | 2 |

ACE = Adaptive designs CONSORT Extension. E&E = explanation and elaboration. SAP = statistical analysis plan.

“X« Y” means original CONSORT 2010 item Y has been renumbered to X;

“X«” means item reordering resulted in new item X replacing the number of the original CONSORT 2010” item X”;

* New items that should only be applied in reference to ACE;

† Modified items that require reference to both CONSORT 2010 and ACE;

‡ Replacement (modified) item that only requires reference to ACE;

§ Item wording remains unchanged in reference to CONSORT 2010 but we expanded the ACE explanatory text to clarify additional considerations for certain adaptive designs. These unchanged items require reference to CONSORT 2010 except for item 14b.

^1^Not applicable since manuscript only reports on protocol, not results.
